# EZH2-miRNA Positive Feedback Promotes Tumor Growth in Ovarian Cancer

**DOI:** 10.3389/fonc.2020.608393

**Published:** 2021-02-25

**Authors:** Ting Liu, Jian Cai, Jing Cai, Zehua Wang, Liqiong Cai

**Affiliations:** Department of Obstetrics and Gynecology, Union Hospital, Tongji Medical College, Huazhong University of Science and Technology, Wuhan, China

**Keywords:** EZH2, H3K27me3, miRNA, ovarian cancer, positive feedback, proliferation

## Abstract

Enhancer of zester homolog 2 (EZH2), a histone methyl transferase that mediates H3K27me3 through polycomb repressive complex 2 (PRC2), is overexpressed in ovarian cancer and promotes malignant proliferation. However, the underlying mechanism of maintaining high EZH2 expression remains elusive. Here we showed that microRNA(miRNA) inhibited EZH2 by binding to the 3′-UTR of EZH2 mRNA; conversely, EZH2 can inhibit miRNA expression. We confirmed that a feedback loop exists between EZH2 and miRNA that maintained EZH2 overexpression, thus promoting ovarian cancer proliferation *in vivo* and *in vitro*. We further explored that EZH2 inhibited miRNA expression through PRC2, as determined by CHIP (chromatin immunoprecipitation), and EZH2 decreased the expression of p21, p53, and RUNX3. These results suggest that EZH2 inhibits the expression of Et-miRNAs (EZH2-targeting miRNAs) through the H3K27me3 pathway, thus forming an EZH2-miRNA positive feedback loop that maintains the high expression of EZH2 and promotes the malignant proliferation of cancer cells by regulating the expression of cell proliferation-related proteins.

## Introduction

Ovarian cancer is the most fatal gynecological cancer. In 2019, it was estimated that 22,530 American women were newly diagnosed with ovarian cancer, and approximately 13,980 women died of this disease (1). Because ovarian cancer develops asymptomatically and rapidly, most patients are diagnosed at a late stage; and the five-year survival rate for late-stage patients is less than 30%, while the survival rate for early-stage patients can be as high as 92% (1). Given that proliferation plays an important role in the development of ovarian cancer, it indicates that the underlying molecular mechanisms should be investigated to aid in the diagnosis and therapy of ovarian cancer.

Enhancer of zeste homolog 2(EZH2), the catalytic subunit of the multiprotein histone methyltransferase complex known as polycomb repressive complex 2 (PRC2) (2), plays an important role in tumorigenesis and tumor progression. It can induce chromatin compaction and subsequent transcriptional silencing by methylating lysine 27 of histone H3(H3K27) (3, 4). EZH2 is highly expressed in a series of cancers, including ovarian cancer, breast cancer, prostate cancer and kidney cancer, and plays an important role in cancer tumorigenesis and progression (5–10). Some findings have shown that EZH2 promots tumor progression through regulating some molecules including p53, p21 and RUNX3, which were related to tumor proliferation (11–15). Our previous study found that EZH2 is highly expressed in ovarian cancer compared with that in benign and borderline ovarian tumors (16). Additionally we found EZH2 is highly expressed in the SKOV3 3rd cell line, a subline of SKOV3 with an expanded stem cell pool established by *in vivo* chemotherapy, and helped to maintain stemness and drug resistance of ovarian cancer stem cells. These findings indicate that EZH2 is a key driver of ovarian cancer; however, the mechanisms underlying its high expression remain unclear.

MicroRNAs (miRNAs) are short (20- to 23-nucleotide), endogenous, single-stranded RNA fragments that regulate gene expression. Mature miRNAs and Argonaute (Ago) proteins form the RNA-induced silencing complex (RISC) which is a ribonucleoprotein complex that complements the 3’UTR of target genes and mediates posttranscriptional gene silencing (17). Studies have shown that miRNA can inhibit cell proliferation, invasion and migration in ovarian cancer. In nasopharyngeal carcinoma, liver cancer and prostate cancer, some microRNAs regulate the development of tumors through EZH2 (18–20). Additionally, in pancreatic ductal adenocarcinoma, EZH2 promoted cell proliferation through inhibiting miR-34a (21). In HCC cells, a reciprocal negative feedback exists between EZH2 and miR-101-1, which inhibited the expression of miR-101-1 and promoted the progression of HCC (22). We proposed that a similar feedback may exist in ovarian cancer to maintain the high expression of EZH2.

In the present study, we proposed and verified a positive feedback loop between EZH2 and miRNAs that maintained a high EZH2 expression in ovarian cancer. We proved that this feedback accelerated the proliferation of ovarian cancer *in vivo* and *in vitro* and then further explored the underlying mechanism of the feedback and its downstream mediators.

## Materials and Methods

### Bioinformatics and Statistical Analysis

To select miRNAs targeting EZH2 in ovarian cancer, we searched miRNAs targeting EZH2 in ENCORI (http://starbase.sysu.edu.cn/) and miRTarBase (http://mirtarbase.mbc.nctu.edu.tw/php/search.php) (**Table S1** and **S2**) and then constructed Venn diagrams to analyze the overlapped regions. The RNA-seq data of ovarian cancer(FPKM values) were downloaded from the Cancer Genome Atlas (TCGA) website (https://cancergenome.nih.gov/) and included five files(gdc_download_20191116_004352.670030.tar, gdc_manifest_20191115_052959, metadata.cart.2019-11-15.json, clinical.cart.2019-11-16.json and clinical.cart.2019-11-16.tar). The miRNA data were downloaded from http://firebrowse.org. We analyzed the correlation between EZH2 and these miRNAs, and different batches were selected (**Table S3**). For correlation analysis, the Pearson correlation coefficient was used. For survival analysis, Kaplan–Meier curves were constructed, and differences between them were analyzed by the log-rank test. A student t test was used for two-sample comparisons. P < 0.05 was considered as statistically significant. IBM SPSS statistics 20, Graphpad Prism 5 and Excel were used for statistical analysis.

### Cell Culture and Transfection

The human ovarian cancer cell lines A2780, SKOV3 and ES2 were obtained from the China Center for Type Culture Collection (China). SKOV3^LV-EZH2^ was constructed by lentivirus with upregulated EZH2, while SKOV3^LV-NC^ was the control. A2780^sg87^ and SKOV3^sg87^ were obtained by CRISPR/Cas9 lentivirus transfection targeting inhibition of EZH2, SKOV3 ^sg87-13#^ was obtained from monoclonal selection using the infinite dilution method, and SKOV3^sgnc^, A2780^sgnc^ were the corresponding control. A2780^sh950^ was obtained by shRNA targeting EZH2, and A2780^shnc^ was the control. Cells were maintained in a humidified incubator with 5% CO_2_ (37°C) in DMEM supplemented with 10% FBS. 3-Deazaneplanocin A (DZNep) and GSK126 were purchased from Cayman Chemical, USA.

### Quantitative Real-Time PCR

Total RNA was extracted with TRIzol (Invitrogen) according to the manufacturer’s instructions. RNA quality and quantity were detected using a NanoDrop 2000/2000C instrument (Thermo Scientific). The primer sequences are listed in **Table S4**. In addition to standard curve analysis, PCRs were performed using SYBR Green Real-time PCR Master Mix (TaKaRa). The two-step PCR program was as follows: 95°C for 30 s, 40 cycles at 95°C for 5 s, 60°C for 30 s, and 70°C for 10 s. The expression of microRNAs was detected by using the stem-loop qRT–PCR assay, and the PCR program was as follows: 95°C for 20 s, 40 cycles at 95°C for 10 s, 60°C for 20 s and 72°C for 10 s. The 2^-ΔΔCt^ method was used to calculate the expression of miRNA and mRNAs at the same time using U6 and ACTIN as internal controls respectivey.

### Western Blotting

Total protein was extracted using NP-40 lysis buffer with cocktail for 10 min. After sonication, the protein lysates were centrifuged at 12,000 rpm for 15 min. After measuring the protein concentrations using the bicinchoninic acid (BCA) assay, the protein extracts were separated by 10% SDS-PAGE and were transferred to a PVDF membrane. After blocking with 5% nonfat milk, the membranes were incubated with primary antibody against β-actin (1:5000; CST, USA), EZH2 (1:2,000; CST, USA), H3K27me3 (1:1,000; Bclonal, Woburn, MA, USA), H3 (1:1,000; ABclonal, Woburn, MA, USA), CHEK1 (1:1,000; CST, USA), P21 (1:1,000; CST, USA), P53 (1:1,000; CST, USA), RUNX3 (1:1,000; CST, USA) at 4°C overnight. Next the membranes were incubated with HRP-conjugated anti-mouse or anti-rabbit secondary antibodies (1:5,000; CST, USA) for 1 h. The protein bands on the membranes were detected using a chemiluminescence kit (Thermo Scientific, MA, USA) and Molecular Imager^®^ ChemiDocTM XRS and Image Lab Software (Bio-Rad Laboratories, Hercules, CA, USA).

### Intraperitoneal Xenograft Model

The animal experiments were supervised and approved by the Animal Care and Use Center of theTongji Medical College, Huazhong University of Science and Technology. Four- to six-week-old female BALB/c nude mice (Beijing Vital River, China) were used to construct the mouse model. MiRNA agomirs were purchased from GenePharma (Suzhou, China). The ovarian cancer cell line SKOV3 stably transfected with LV-NC or LV-EZH2 was used for cancer xenografts. Thirty nude mice were randomly divided into two groups; 15 mice in each group were subcutaneously injected with tumor cells. One week after the injection, the nude mice in each group were randomly divided into three groups and were administered agomir nc, agomir miR-101 or agomir miR-26a by intratumoral injection. Agomir dry reagent was diluted with 120 μl of sterile DEPC-treated water. Each subcutaneous tumor was injected with 80μl of miRNA agomir three times once a week, and the body weight and tumor volume of the nude mice were measured every 3 days. All the mice were killed 5 weeks after tumor inoculation. All tumors were removed, weighed and measured in volume. The tumor was divided into two parts: one part was embedded in paraffin for H-E staining and histopathological analysis of EZH2 and Ki67, while the other part was cryopreserved at −80°C.

### Chromatin Immunoprecipitation Assay

CHIP assay was performed based on the manufacturer’s protocol of the EpiQuik CHIP kit (Epigentek, Farmingdale, NY, USA). After cell formaldehyde fixation and lysis, chromatin was fragmented to ~200−1,000 bp. Next the primary antibodies anti-EZH2(CST, USA) and anti-H3K27me3 (ABclonal, Woburn, MA, USA) were used for coimmunoprecipitation. Additionally, mouse IgG and anti-H3 antibody in the kit were used as negative and positive controls respectively. The extracted DNA was used for RT–PCR, and the primers for CHIP are listed in **Table S5**.

### Clonal Formation Assay

Forty-eighty hours after treatment, the cells were inoculated into a six-well plate, each well containing 500 cells. After culture in DMEM containing 10% serum for 2 weeks, the number of colonies was counted. Each experiment was repeated three times under the same condition and the data were reported as the ratio between treated and control groups.

### 5-Ethynyl-20-Deoxyuridine Proliferation Assay

The assay was performed according to the manufacturer’s instructions of the EdU experiment kit (RiboBio, Guangzhou, China). EdU (5-ethynyl-20-deoxyuridine) is a nucleoside analog of thymine that can be integrated into DNA during DNA synthesis. The cells were inoculated into a 96-well plate at a density of 6,000 cells per well. After 24 h, 100 μl of 0.1% EdU reagent was added to each well for 2 h, followed by fixation with 4% formaldehyde at room temperature for 30 min. After treatment with 0.5% Triton for 10 min, the cells were incubated with 1×Apollo^®^ reagent for 30 min. Finally, Hoechst 33342 was added to counterstain cells at room temperature for 30 min. The proportion of EdU-bound cells was the proportion of proliferating cells. The results were obtained from three independent repeated experiments.

### Immunohistochemistry

The nude mouse tumor was submitted to the company to be sliced. First, the slices were placed in the oven and baked for 2 h. After dewaxing, the slices were placed into TO-I solution, TO-II solution, TO-III solution, anhydrous ethanol, 95% alcohol, 75% alcohol, 50% alcohol and double distilled water in turn. Next, the slices were incubated in the antigen repair solution. To inactivate the endogenous peroxidase and biotin of the tissue, the slices were incubated in 0.3% hydrogen peroxide for approxinately 20 min. Goat serum was added to block the tissue for 1 h. The tissue slices were incubated with the rabbit anti-EZH2 antibody (1:150 dilution, CST) or rabbit anti-Ki67 antibody (1:250 dilution, Abcam) overnight at 4°C. After washing with PBS the next day, the tissue slices were incubated with the secondary antibody for 30 min at room temperature and then with horseradish peroxidase for 20 min. DAB dye solution was prepared (DAB 20 × concentrated original solution and DAB diluent were prepared at 1:20), and two drops of DAB working solution were dripped into the tissue area of each tablet. Under the microscope, a brown appearance indicated positive staining. The tissue slices were rinsed gently under running water for 15 min. Next, the tissue slices were stained with hematoxylin and then were hydrated in double-distilled water, 50% alcohol, 75% alcohol, 95% alcohol, and anhydrous ethanol. The tissue slices were then sealed and dried in an oven, followed by photo observation and score analysis. The discrimination of the expression of objective factors was mainly based on the depth of staining and proportion of stained cells. The degree of staining was classified as follows: 0 points, no color or unclear color; 1 point, light yellow; 2 points, brownish yellow; 3 points, dark brown. The proportion of stained cells was classified as follows: 0, 0–1%; 1, 25%; 2, 26–50%; 3, 51–75%; 4, 76–100%. The immunohistochemical score comprised the depth of staining and product of the stained cells. At least 5 high power visual fields were randomly observed during the scoring process.

## Results

### Identification of Et-miRNAs in Ovarian Cancer

**Figure 1:**
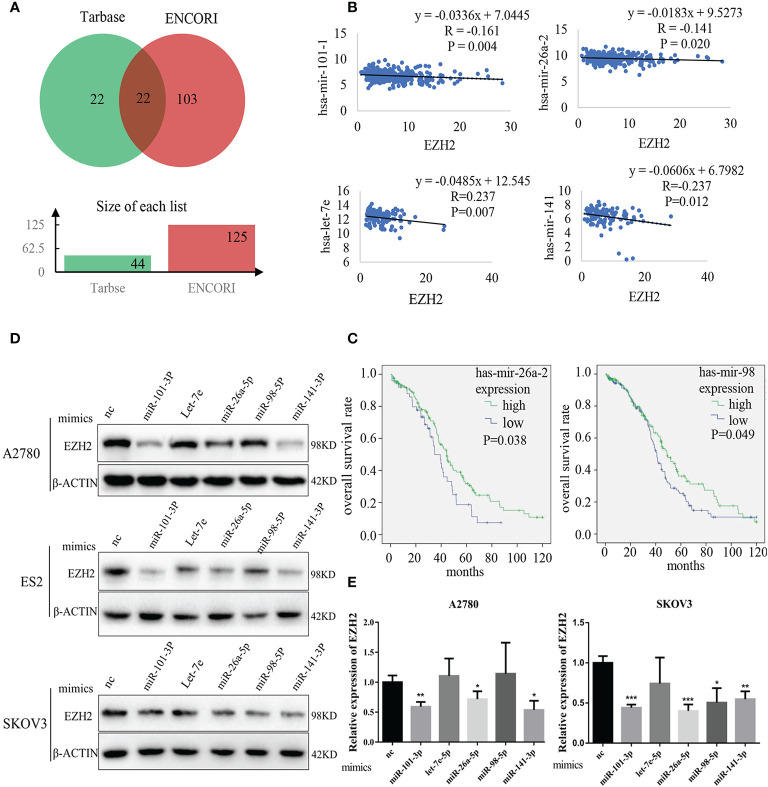
Identification of Et-miRNAs in ovarian cancer. **(A)** Venn diagram of miRNAs targeting EZH2 mRNA predicted by Tarbase and ENCORI. **(B)** Correlation analysis of miRNAs and EZH2 expression in ovarian cancers using data from TCGA. The ordinate scales show the expression of miRNAs in $\log_{2}^{\left(\text{x}+1\right)}$. **(C)** Kaplan–Meier curves of overall survival in patients with ovarian cancer, samples from TCGA. The differences between groups were analyzed by the log-rank test. Censored data are indicated by tick marks. **(D)** Representative images of western blot assays for EZH2 protein in A2780, ES2 and SKOV3cell lines after the transfection of Et-miRNA mimics. **(E)** qRT–PCR for EZH2 mRNA in A2780 and SKOV3 cells after transfection with miRNA mimics. *P < 0.05, **P < 0.01, ***P < 0.001.

The potential EZH2-targeting miRNAs (abbreviated as Et-miRNAs) were initially investigatedly using two tools to predict miRNA-mRNA interactions, ENCORI and miRTarBase. Twenty-two miRNAs were identified that might interact with EZH2 mRNA were predicted by both tools (**Figure 1A** and **Tables S1** and **S2**). By analyzing samples from the TCGA database, we found that hsa-mir-101-1, hsa-mir-26a-2, and hsa-let-7e were negatively associated with EZH2 expression in ovarian cancer. Additionally, hsa-miR-141, a miRNA that has been reported to target EZH2 in prostate cancer, was included because of its significant correlation with EZH2 in ovarian cancer (**Figure 1B**). Moreover, patients with higher expression levels of hsa-mir-26a-2 and hsa-mir-98 showed better overall survival (**Figure 1C** and **Table S3**). To confirm that Et-miRNAs can inhibit EZH2 in ovarian cancer, the expression levels of the above five Et-miRNAs and EZH2 in the A2780, SKOV3, and ES2 cell lines were examined (**Figures S1A, B**). After the transfection of miR-101-3p mimics, miR-26a-5p mimics and miR-141-3p mimics, the EZH2 protein and mRNA levels were reduced in A2780, ES2, and SKOV3 cell lines(**Figures 1D, E**).

### Enhancer of Zester Homolog 2 Inhibits Et-miRNAs Through H3K27 Trimethylation

**Figure 2:**
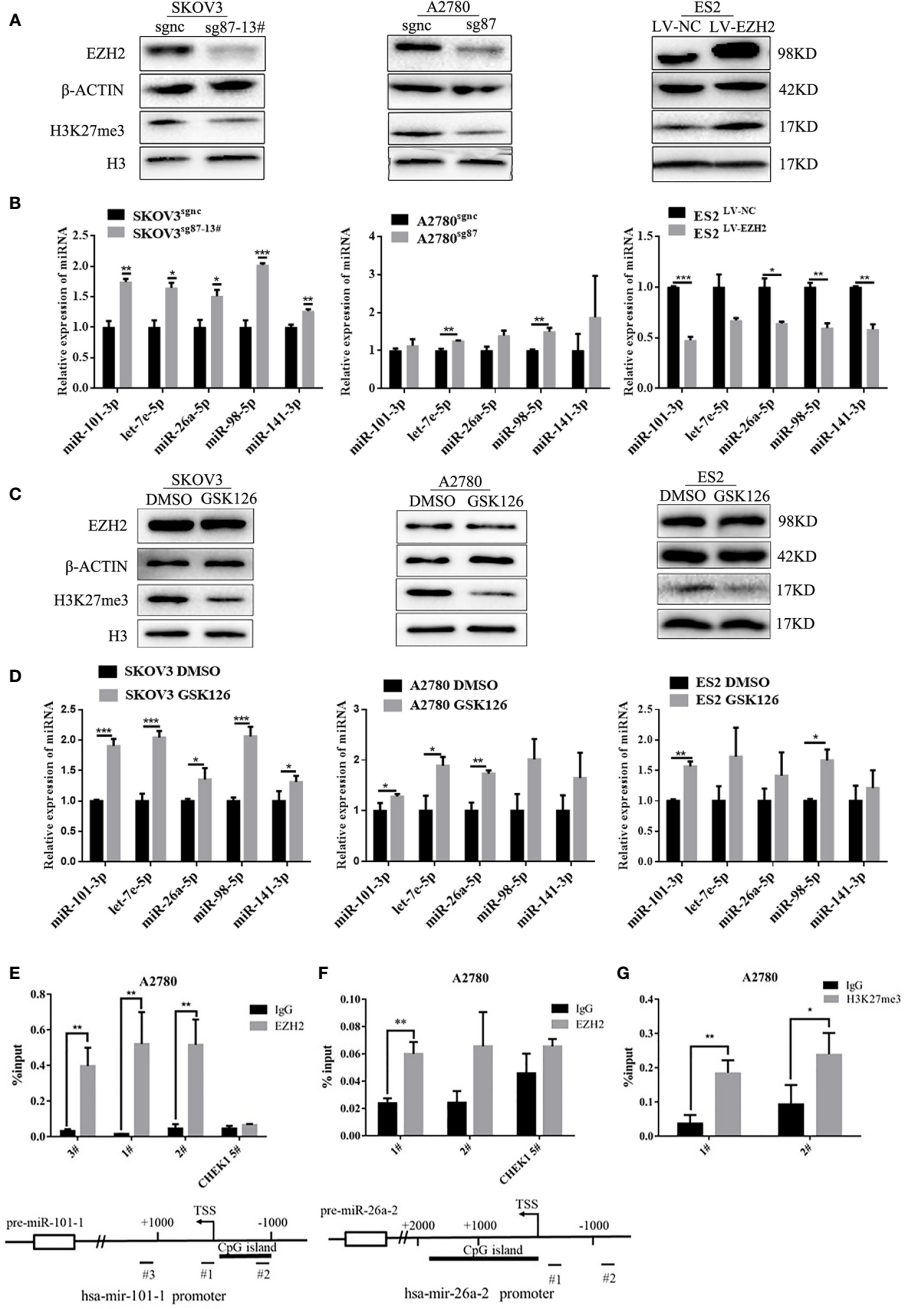
EZH2 inhibits Et-miRNAs through H3K27 trimethylation. **(A)** Representative images of western blot assays for the EZH2 protein and H3K27me3 levels in SKOV3 ^sg87-13#^, A2780^sg87^ and ES2^LV-EZH2^. **(B)** Expression of Et-miRNAs in SKOV3^sg87-13#^, A2780^sg87^ and ES2^LV-EZH2^ as detected by qRT–PCR. **(C)** Representative images of western blot assays for EZH2, H3 protein and the level of H3K27me3 in ovarian cancer cells with/without GSK126 treatment for 48h. **(D)** qRT–PCR for the expression of Et-miRNA in ovarian cancer cells with/without GSK126 treatment for 48h. **(E)** Location of the binding site of primers and the result of CHIP–qPCR for hsa-mir-101-1 and miR-101-3p can be generated from the two pre-miRNAs hsa-mir-101-1/hsa-mir-101-2 encoded by two different DNA sequences. **(G)** Location of the binding site of primers and the result of CHIP–qPCR for hsa-mir-26a-2 and miR-26a-5p can be generated from two pre-miRNAs hsa-mir-26a-1/hsa-mir-26a-2 encoded by two different DNA sequences. **(F)** The result of CHIP–qPCR with H3k27me3 antibody for hsa-mir-101-1. Primer CHEK1 5#, a primer targeting the CHEK1 promoter region that is not affected by EZH2, was used as a negative control. TSS, transcription start site. *P < 0.05, **P < 0.01, ***P < 0.001.

To investigate the effect of EZH2 on Et-miRNAs, the CRISPR/Cas9 technique was used to silence EZH2 in A2780 and SKOV3 cells(A2780^sg87^ and SKOV3^sg87^ obtained) and a monoclonal cell line (SKOV3^sg87-13#^) was obtained using the infinite dilution method. Additionally, we upregulated EZH2 by transfecting lentivirus harboring the EZH2 overexpression plasmid in ES2 and SKOV3 cells(ES2^LV-EZH2^ and SKOV3^LV-EZH2^ were obtained). We found that EZH2 expression in A2780^sg87^ and SKOV3^sg87^ was reduced by 80–90% compared with that in negative control cell, accompanied by decreased H3K27me3 levels and upregulation of hsa-miR-101-3p, has-let-7e-5p, hsa-miR-26a-5p, hsa-miR-98-5p, and hsa-miR-141-3p (**Figures 2A, B**). By contrast, H3K27me3 increased and Et-miRNAs were downregulated in ES2^LV-EZH2^ and SKOV3^LV-EZH2^ compared with those in the control group (**Figures 2A, B** and **Figure S2A**). Following treatment of ovarian cancer cells with GSK126 to inhibit the methyltransferase activity of EZH2, no significant change was found in the expression of EZH2 in ovarian cancer cells compared with that in the control group, the level of H3K27me3 was decreased, and the expression of Et-miRNAs was upregulated (**Figures 2C, D**). These results indicate that EZH2 can inhibit Et-miRNA’ expression, while the mechanism remains unknown. To explore whether EZH2 regulates Et-miRNAs through histone H3K27 trimethylation on the Et-miRNA promoter, we carried out the CHIP assay near the transcription start site (TSS) on the Et-miRNA promoter region using the EZH2 and H3K27me3 antibodies in the ovarian cancer cell line A2780. According to the UCSC Genome browser, the Et-miRNA’ promoter is rich in CpG islands and three pairs of primers were designed for each Et-miRNA. EZH2 was enriched in the promoter region of Et-miRNAs (**Figures 2E, F** and **Figure S2B–F**), and H3K27me3 was enriched at the miR-101-1 promoter (**Figure 2G**). These results suggest that EZH2 suppresses Et-miRNAs through H3K27 trimethylation on the promoter.

### Enhancer of Zester Homolog 2-miRNA-Loop Sustains High Enhancer of Zester Homolog 2 Expression and Proliferation Capacity

**Figure 3:**
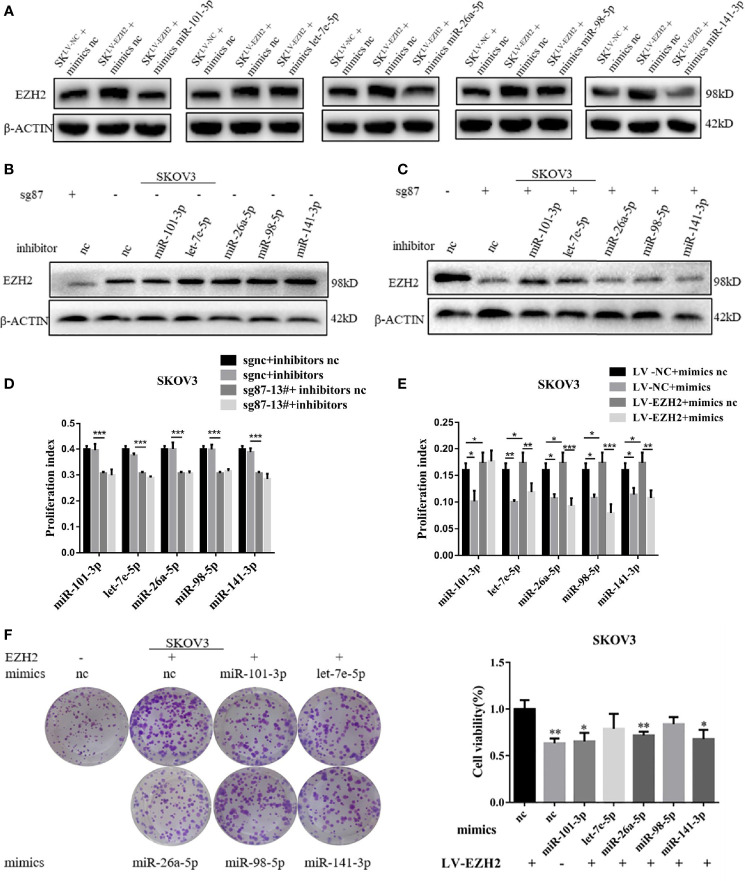
The EZH2-miRNA-loop sustains high EZH2 expression and proliferation capacity. **(A)** Representative images of western blot assays for EZH2 protein in SKOV3^LV-NC^ and SKOV3^LV-EZH2^ cells transfected with Et-miRNA mimics. **(B, C)** Representative images of western blot assays for EZH2 protein in SKOV3^sgnc^ and SKOV3^sg87^ cells transfected with Et-miRNA inhibitors. **(D)** EdU assay to detect the cell proliferation ability of SKOV3^LV-EZH2^ and SKOV3^LV-NC^ cells transfected with Et-miRNA mimics. **(E)** EdU assay to detect the cell proliferation ability of SKOV3^sg87-13#^ and SKOV3^sgnc^ cells transfected with Et-miRNA inhibitors. **(F)** Cell clone formation assay for SKOV3^LV-EZH2^ cell transfected with Et-miRNA mimics. *P < 0.05, **P < 0.01, ***P < 0.001.

**Figure 4:**
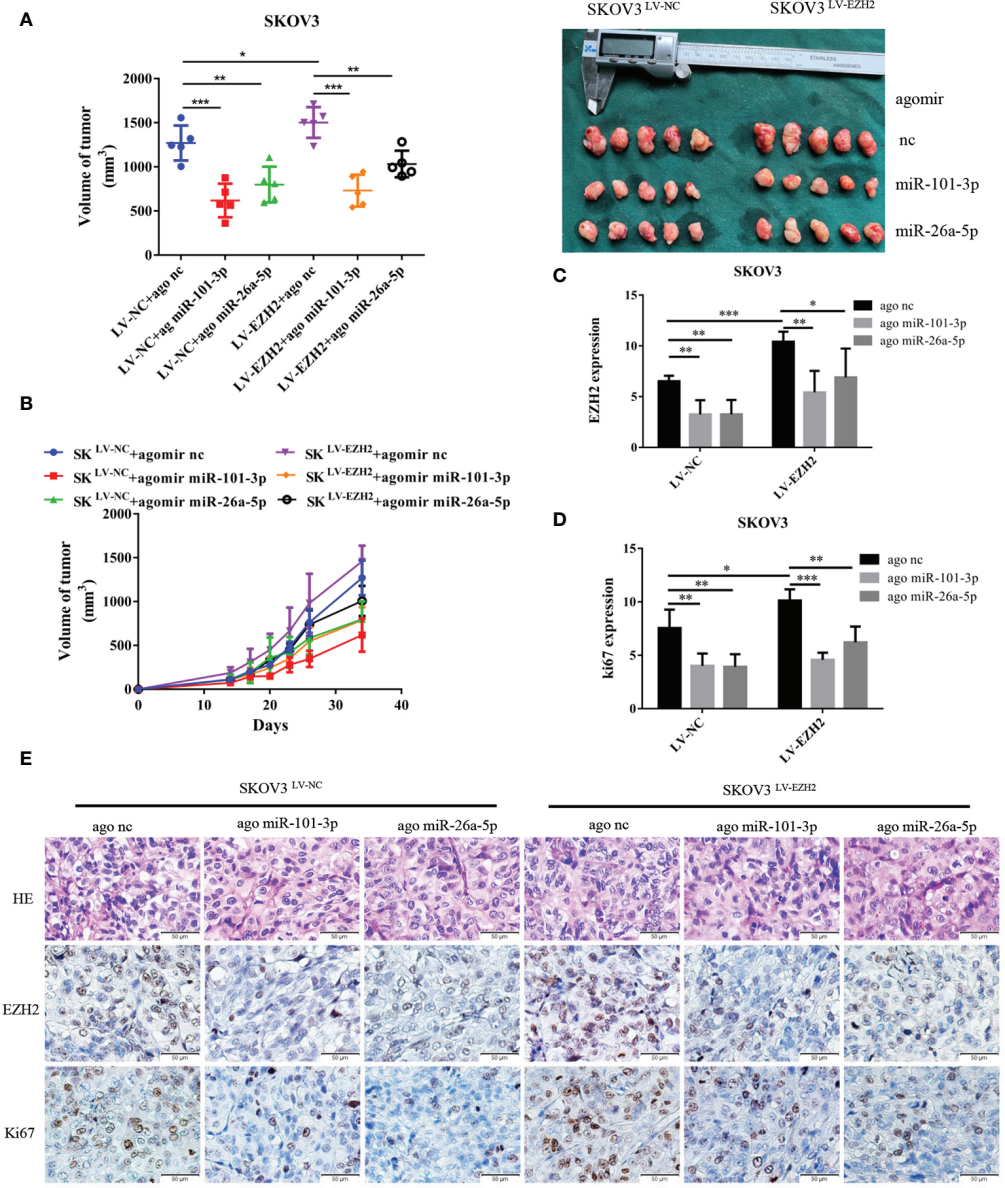
Et-miRNAs inhibit SKOV3-xenograft growth. Nude mice were randomly divided into six groups: SKOV3^LV-NC^+ agomir nc group, SKOV3^LV-NC^+ agomir miR-101 group, SKOV3^LV-NC^+ agomir miR-26a group, SKOV3^LV-EZH2^+ agomir nc group, SKOV3^LV- EZH2^+ agomir miR-101 group, SKOV3^LV-EZH2^+ agomir miR-26a group. **(A, B)** Tumor volume growth curves for different treatment groups and subcutaneous tumor volume in nude mice. **(C–E)** HE staining of subcutaneous tumor tissue sections and representation of the immunohistochemistry of EZH2, cell proliferation marker Ki67 and statistical diagram of the results. The scale bar length represents 50 microns in **(E)**. *P < 0.05,**P < 0.01,***P < 0.001.

To confirm the positive feedback loop between EZH2 and Et-miRNAs and whether the feedback could maintain high EZH2 expression in ovarian cancer, Et-miRNAs expression in SKOV3^sg87-13#^ and SKOV3^LV-EZH2^ cancer cells were manipulated by transfection with miRNA inhibitors or mimics. After transfection with miR-101-3p mimics, miR-26a-5p mimics and miR-141-3p mimics, the EZH2 protein level was reduced in SKOV3^LV-EZH2^ (**Figure 3A**). On the other hand, the expression of EZH2 protein was increased in SKOV3^sgnc^ cells transfected with Et-miRNA inhibitors (**Figure 3B**). However, after transfection with Et-miRNA inhibitors, no significant change was noted in the expression of EZH2 in SKOV3^sg87^ (**Figure 3C**). Considering that EZH2 was downregulated obviously, the effect of inhibitors on EZH2 seemed inconspicuous. These results indicated that the positive feedback loop between EZH2 and miRNA could maintain EZH2 high expression in ovarian cancer. Next, we manipulated Et- miRNA expression in A2780, ES2, SKOV3 cancer cells by transfection with miRNA mimics or inhibitors and found that cell proliferation was promoted by the miRNA inhibitors and inhibited by the miRNA mimics using the EdU assay (**Figures S3A–D**). Additionally, miRNA mimics were transfected into SKOV3^LV-NC^/SKOV3^LV-EZH2^, and miRNA inhibitors were transfected into SKOV3^sgnc^/SKOV3^sg87-13#^. EdU proliferation and clone formation assays showed that cell proliferation was significantly enhanced in SKOV3 with EZH2 upregulation, which could be partially relieved by Et-miRNA mimics (**Figures 4D, F** and **S3E**). Additionally in SKOV3 ^sg87-13#^, cell proliferation was inhibited compared with the control group, but this impairment could not be reversed by inhibitors (**Figures 4E** and **S3F**).To explore how the high expression of EZH2 caused by the EZH2-miRNA positive feedback promotes the malignant proliferation of ovarian cancer, we examined the expression of the proliferation-related molecules p21, p57, p27, p53, E2F1, cyclin d1, c-myc and RUNX3, which function downstream of EZH2 by qRT–PCR. Because the variations of p27, c-myc and E2F1 in SKOV3, SKOV3 ^LV-NC^ and SKOV3^sgnc^ were large, further experiments were carried out without them. qRT-PCR and western blot assay showed that the expression of p21, p53 and RUNX3 was downregulated in SKOV3^LV-EZH2^ cells and upregulated in SKOV3 ^sg87-13#^ and A2780^sh950^ cells (**Figures S3G–I**). After treatment with DZNep, an inhibitor of EZH2, the expression of EZH2 protein was decreased and of p21, p53 and RUNX3 was increased in ovarian cancer cells A2780, ES2 and SKOV3 (**Figure S3J**). These results indicated that EZH2 promotes the malignant proliferation of ovarian cancer cells by regulating the expression of proliferation-related proteins.

### Et-miRNAs Inhibit SKOV3 Xenograft Growth

We established xenografts by subcutaneous injection of SKOV3^LV-NC^ and SKOV3^LV-EZH2^ cells into nude mice and intratumoral injections of agomir nc, agomir miR-101-3p or agomir miR-26a-5p once a week for three cycles. As anticipated, tumor grew more rapidly with the administration of SKOV3 ^LV-EZH2^ cells compared with SKOV3 ^LV-NC^ cells. After 30 days of tumorigenesis, the volume of SKOV3^LV-EZH2^ tumors was larger than that of SKOV3^LV-NC^ tumors. Compared with agomir nc, agomir miR-101-3p and agomir miR-26a-5p partially counteracted the proliferation promoting effect of EZH2 (**Figures 4A, B**). Moreover, immunohistochemistry showed that the expression of EZH2 and Ki67 in SKOV3^LV-EZH2^ tumors was higher than that in SKOV3^LV-NC^ (**Figures 4D, E**). After treatment with agomir miR-101-3p or agomir miR-26a-5p, the expression of EZH2 and Ki67 in SKOV3^LV-EZH2^ tumors decreased (**Figure 4C**). These data suggest that inhibition of the EZH2-miRNA positive feedback loop can effectively retard the proliferation of ovarian cancer.

## Discussion

Accumulating evidence has demonstrated the important role of high EZH2 expression in the malignant progression of epithelial ovarian cancer (23, 24), but the underlying regulatory mechanism remains largely unknown and warrants further investigation. In the present study, we found that Et-miRNAs inhibited the expression of EZH2 and proliferation of cancer cells, while reduction of EZH2 caused the increase in Et-miRNA. CHIP assay indicated that EZH2 could be enriched in the promoter region of Et-miRNAs and inhibit the expression of Et-miRNAs through the H3K27me3 pathway. The positive feedback loop between EZH2 and miRNAs maintains the high expression of EZH2, thus promoting the malignant proliferation of ovarian cancer by regulating proliferation-related proteins.

MiRNA has been reported to function as a tumor suppressor in various cancers by targeting multiple oncogenes, including EZH2, and it can inhibit the expression of mRNA by binding to the 3’UTR region of the downstream target gene to suppress translation or degrade mRNA. Let-7a inhibits cell proliferation and apoptosis by inhibiting EZH2 in nasopharyngeal carcinoma cells (25). MiR-138 inhibits tumor growth by inhibiting EZH2 in non-small cell lung cancer (26). In the present study, we found that Et-miRNA can inhibit the expression of EZH2 and impair the proliferation of ovarian cancer *in vivo* and *in vitro*. Additionally, the mechanism of Et-miRNA inhibiting EZH2 was confirmed by double luciferase report assay in other reports.

As a methyltransferase component of the PRC2, EZH2 can catalyze the trimethylation of histone H3 at lysine 27 (H3K27me3), which silences specific gene transcription (27). EZH2 also interacts directly with DNA methyltransferase (DNMT1, DNMT3A, DNMT3b) in a PRC2-dependent manner, mediating chromatin compaction (28). In this study, we found that EZH2 was enriched in the promoter regions of five Et-miRNAs and inhibited their expression. GSK126 decreased the level of H3K27me3 and increased miRNA expression in ovarian cancer cells. Therefore, we concluded that EZH2 inhibited the expression of Et-miRNAs by catalyzing H3K27me3. Similar studies have been reported in other tumors. EZH2 can inhibit the expression of miR-34a by mediating the H3K27 methylation and DNA methylation in bile duct carcinoma (29). In hepatocellular carcinoma, EZH2 can inhibit the expression of miR-22 through H3K27me3 pathway (30). In multiple myeloma, EZH2 participates in drug resistance by regulating H3K27me3 to inhibit miR-38 (31).

It has been reported that a feedback loop exists between EZH2 and miRNA in many tumors. The EZH2/miR-26 feedback loop can regulate tumor growth in hepatocellular carcinoma (32). In multiple myeloma, a feedback loop exists between EZH2/miR-101 (33). However, no similar study has been reported in ovarian cancer. In this study, we demonstrated a positive feedback between EZH2 and miRNAs in epithelial ovarian cancer that promoted malignant proliferation by maintaining the high expression of EZH2. MiRNA mimics could abrogate EZH2-induced cell proliferation. MiRNA inhibitors had no obvious effect on the proliferation of SKOV3^sg87-13#^. The causes may be that, in SKOV3^sg87-13#^, EZH2 was knocked down, causing a sharp decrease in the miRNA target. Because of the high expression of EZH2, the increase in EZH2 expression induced by the miRNA inhibitor had no significant effect on the proliferation of ovarian cancer cells. Thus, that the effect of Et-miRNA inhibitors was not obvious may be that the basic expression of Et-miRNAs in ovarian cancer cells was low.

EZH2 can affect cell proliferation by regulating key growth inhibitors (34). P53 is an important tumor inhibitor involved in the regulation of the cell cycle, apoptosis and DNA damage repair (35–37). P21 is a cyclin-dependent kinase inhibitor downstream of the p53 gene. The synergistic effect of p21 and p53 leads to G1 checkpoint arrest of cell cycle, thus playing an anti-tumor effect (13–15). In HCC, EZH2 regulates p53 through the H3K27me3 pathway (38). In breast cancer, gastric cancer and hepatocellular carcinoma, EZH2 inhibits the expression of p21 by catalyzing H3K27me3 (38–40). RUNX3 is a transcription factor that participates in the regulation of p21. EZH2 also inhibits the expression of RUNX3 in bile duct carcinoma and laryngeal carcinoma (12, 41). In the present study, we found a significant correlation between the expression of p21/p53/RUNX3 and EZH2 in ovarian cancer: downregulation or inhibition of EZH2 could increase the expression of p21, p53, and RUNX3, and upregulation of EZH2 caused decreased expression. EZH2 inhibited these proliferation-related genes to promote the proliferation of ovarian cancer.

In conclusion, our data suggest a positive feedback loop exists between EZH2 and miRNAs in epithelial ovarian cancer that maintains high EZH2 expression and promotes tumor cell proliferation by regulating cell proliferation-related proteins. Our findings provide new insights into the regulatory mechanism between EZH2 and miRNAs in ovarian cancer and some evidence for the application of miRNA related agents and EZH2 inhibitors in ovarian cancer treatment.

## Data Availability Statement

The original contributions presented in the study are included in the article/**Supplementary Material**. Further inquiries can be directed to the corresponding authors.

## Ethics Statement

The animal study was reviewed and approved by Institutional Animal Care and Use Committee Tongji Medical College, Huazhong University of Science and Technology.

## Author Contributions

TL, JAC, JC, ZW, and LC conceptualized and designed the experiment. TL, JAC, JC, LC and ZW developed the methodology. TL and JAC were in charge of the data acquisition (provided animals, cell experiment, etc.). TL and JAC analyzed and interpreted the data (statistical analysis, biostatistics, etc.). TL, JAC, JC, LC and ZW wrote, reviewed and revised the article. JC, ZW, and LC performed process supervision. TL and JAC contributed equally to the article. All authors contributed to the article and approved the submitted version.

## Conflict of Interest

The authors declare that the research was conducted in the absence of any commercial or financial relationships that could be construed as a potential conflict of interest.
