# Supplementary figures and images for: EZH2-miRNA Positive Feedback Promotes Tumor Growth in Ovarian Cancer
Source: Front Oncol. 2021 Feb 25;10:608393. doi: 10.3389/fonc.2020.608393 (PMC7947696; doi:10.3389/fonc.2020.608393)

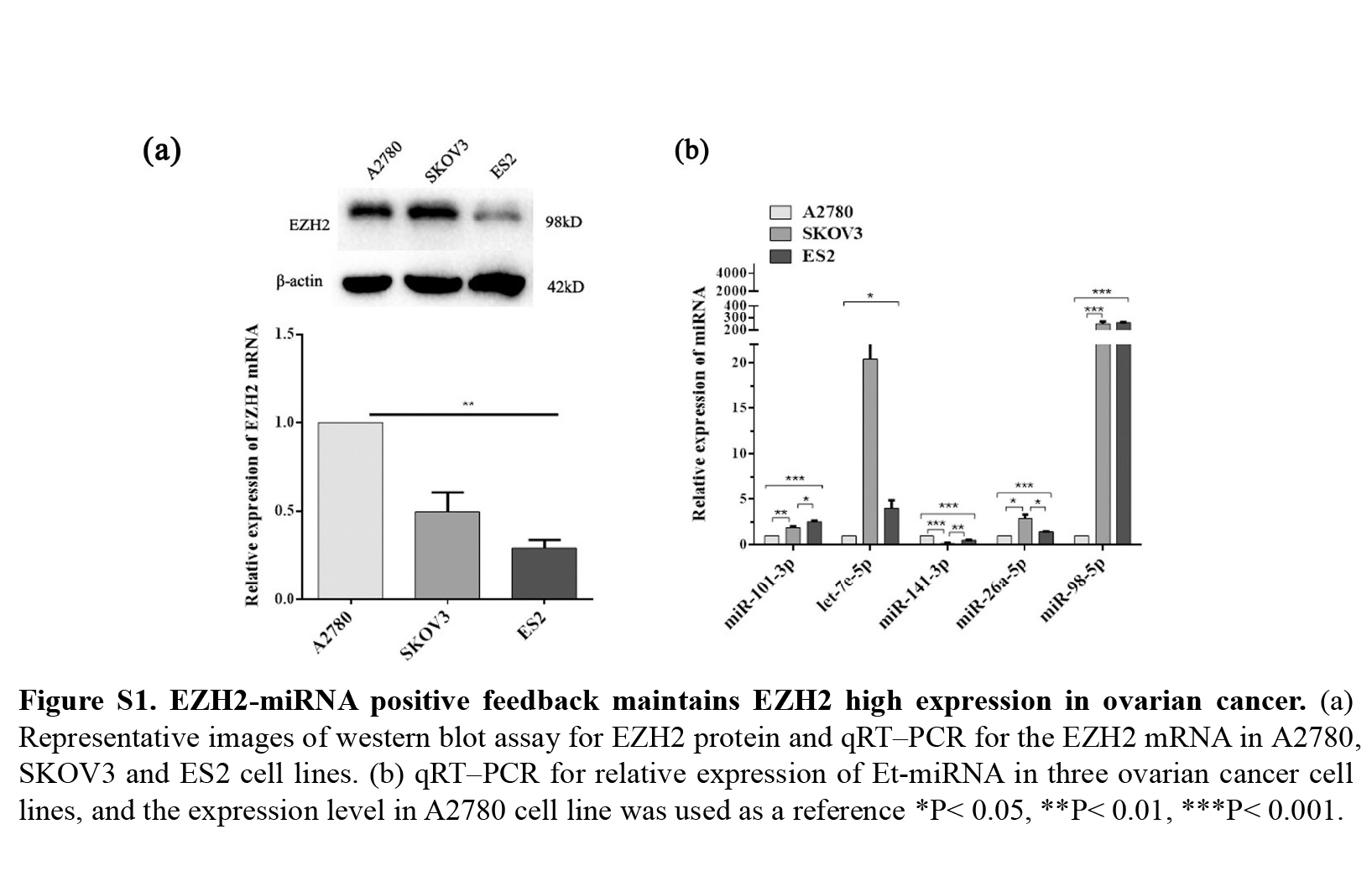

Supplement: Supplementary file 1 [file Image_1.jpeg]

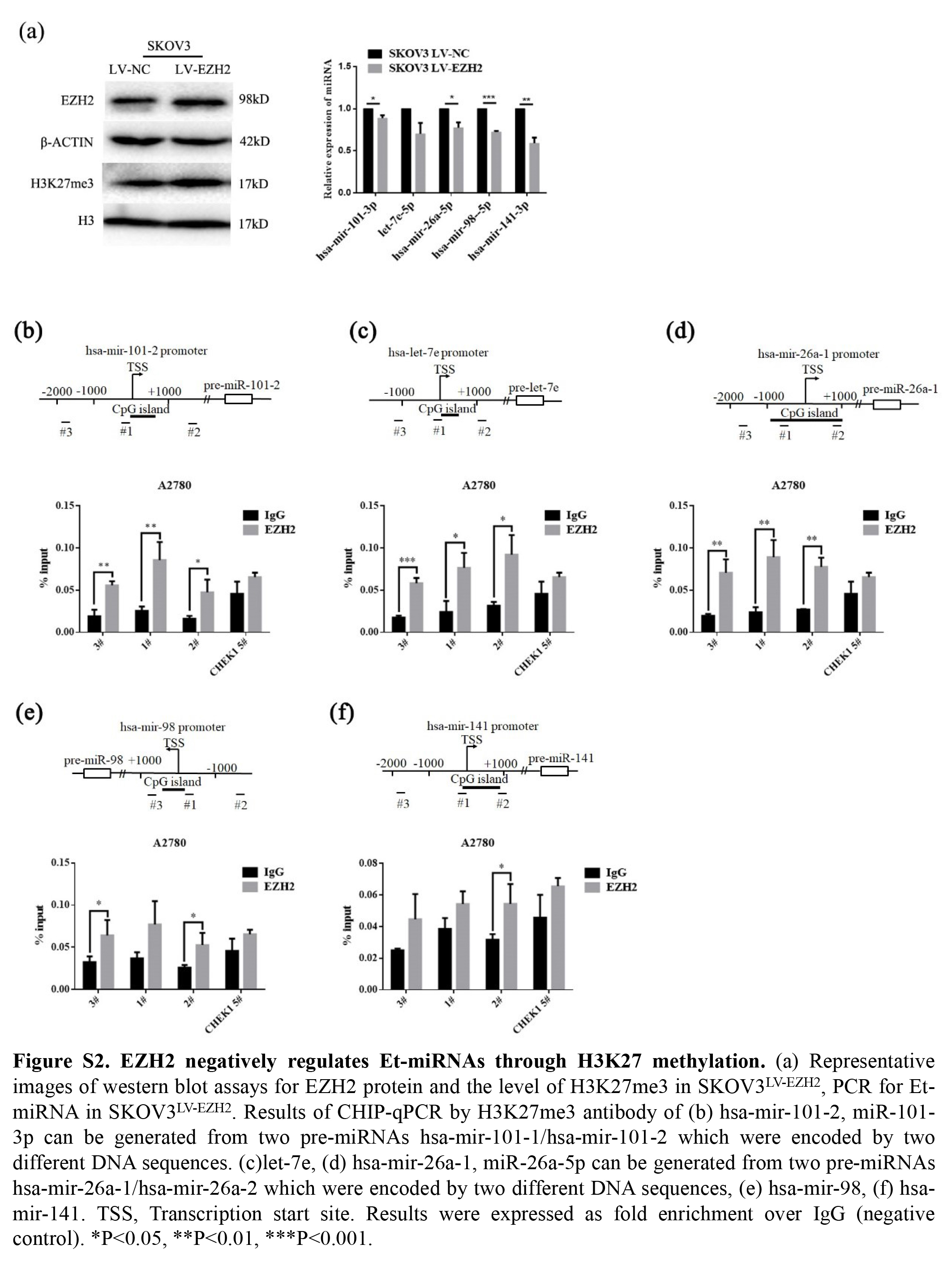

Supplement: Supplementary file 2 [file Image_2.jpeg]

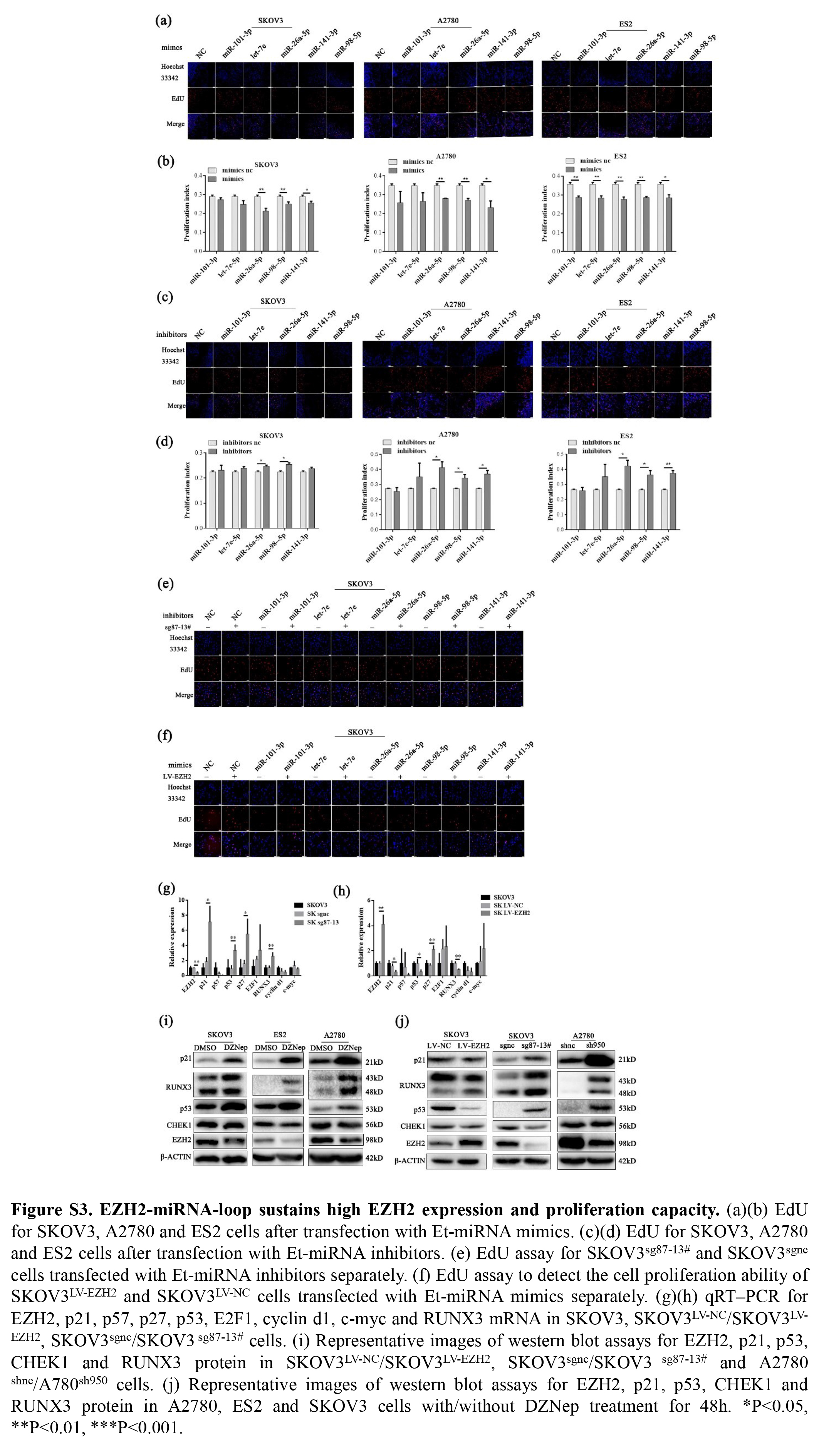

Supplement: Supplementary file 3 [file Image_3.jpeg]

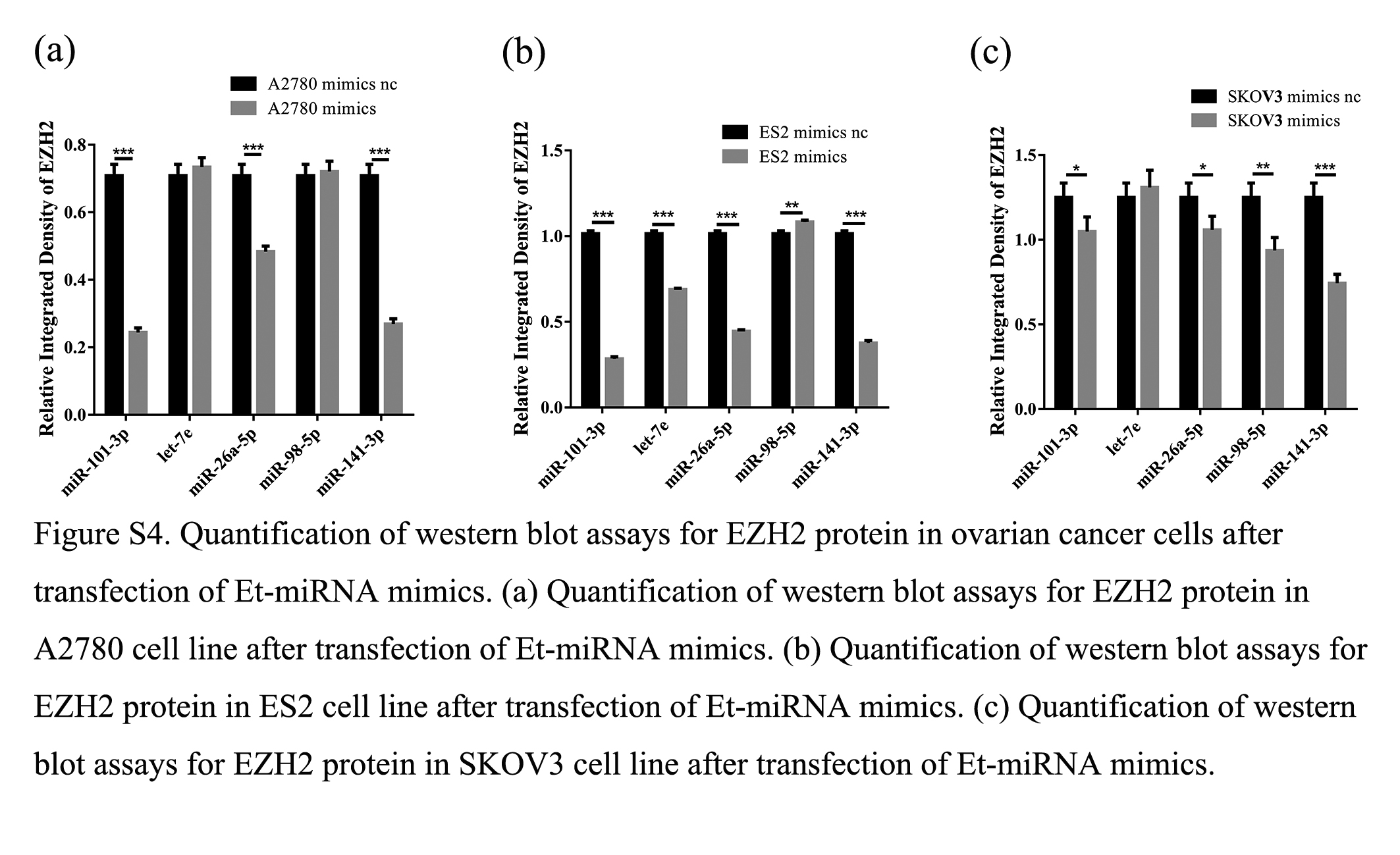

Supplement: Supplementary file 4 [file Image_4.jpeg]

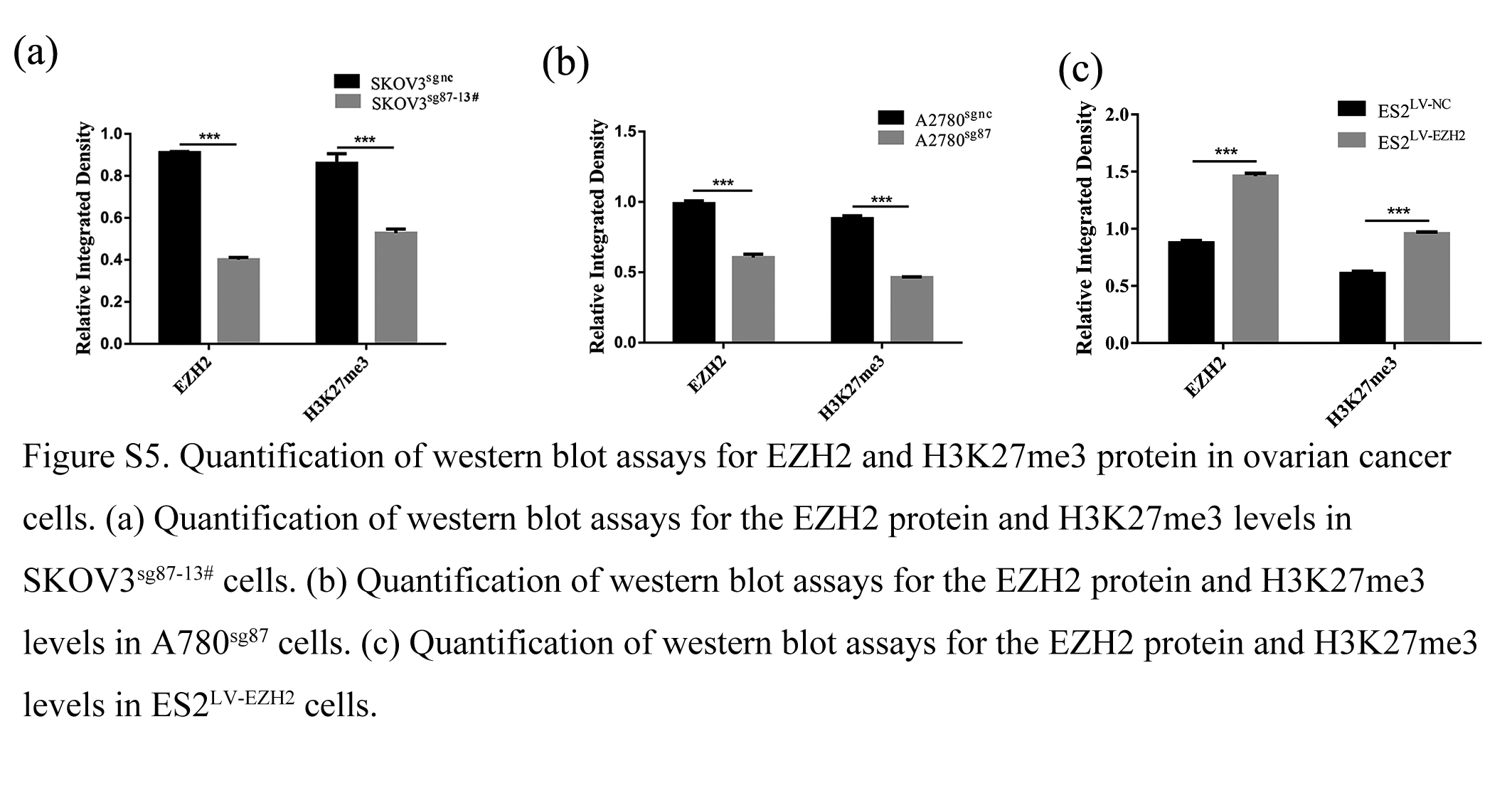

Supplement: Supplementary file 5 [file Image_5.jpg]

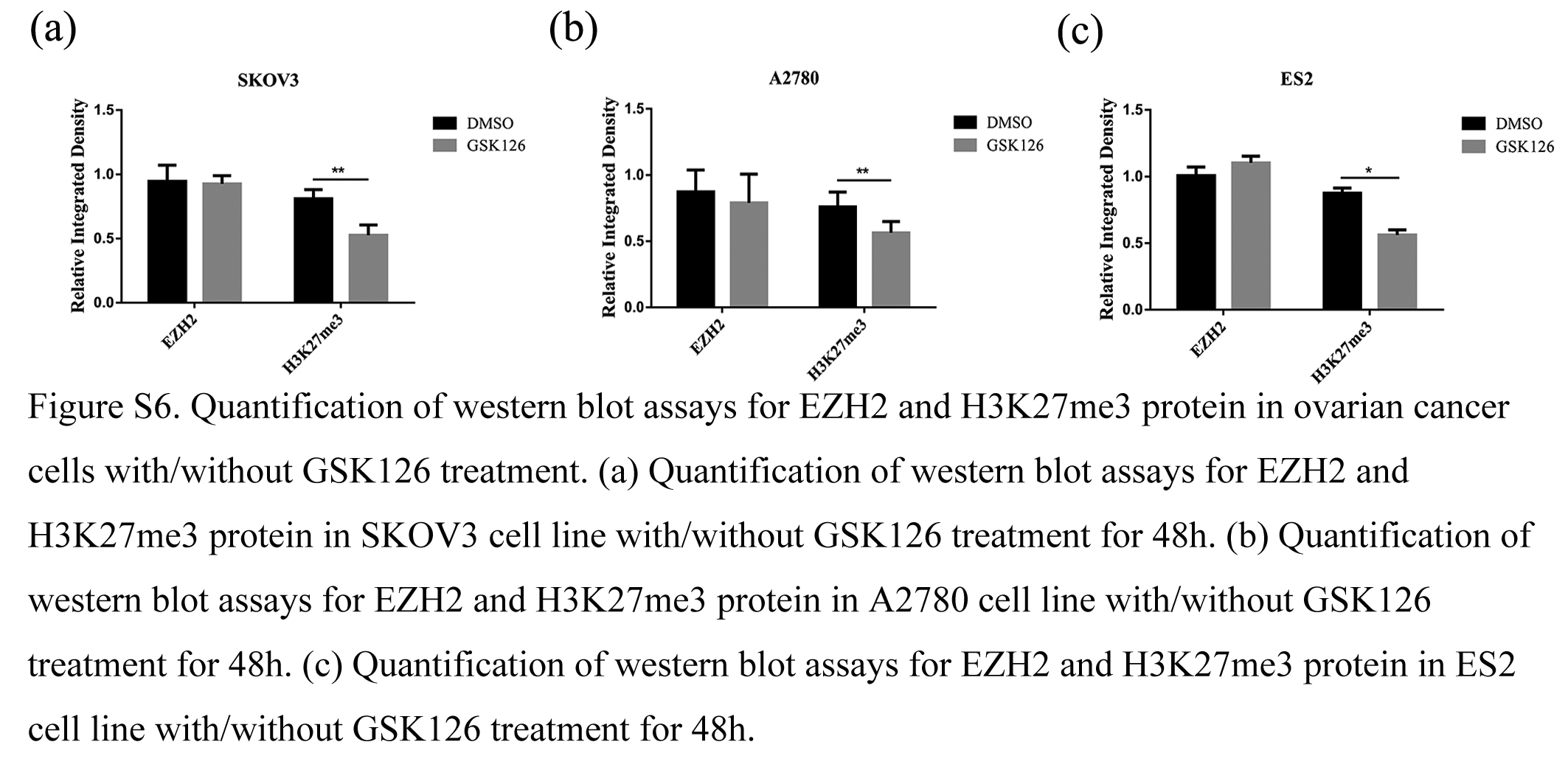

Supplement: Supplementary file 6 [file Image_6.jpeg]

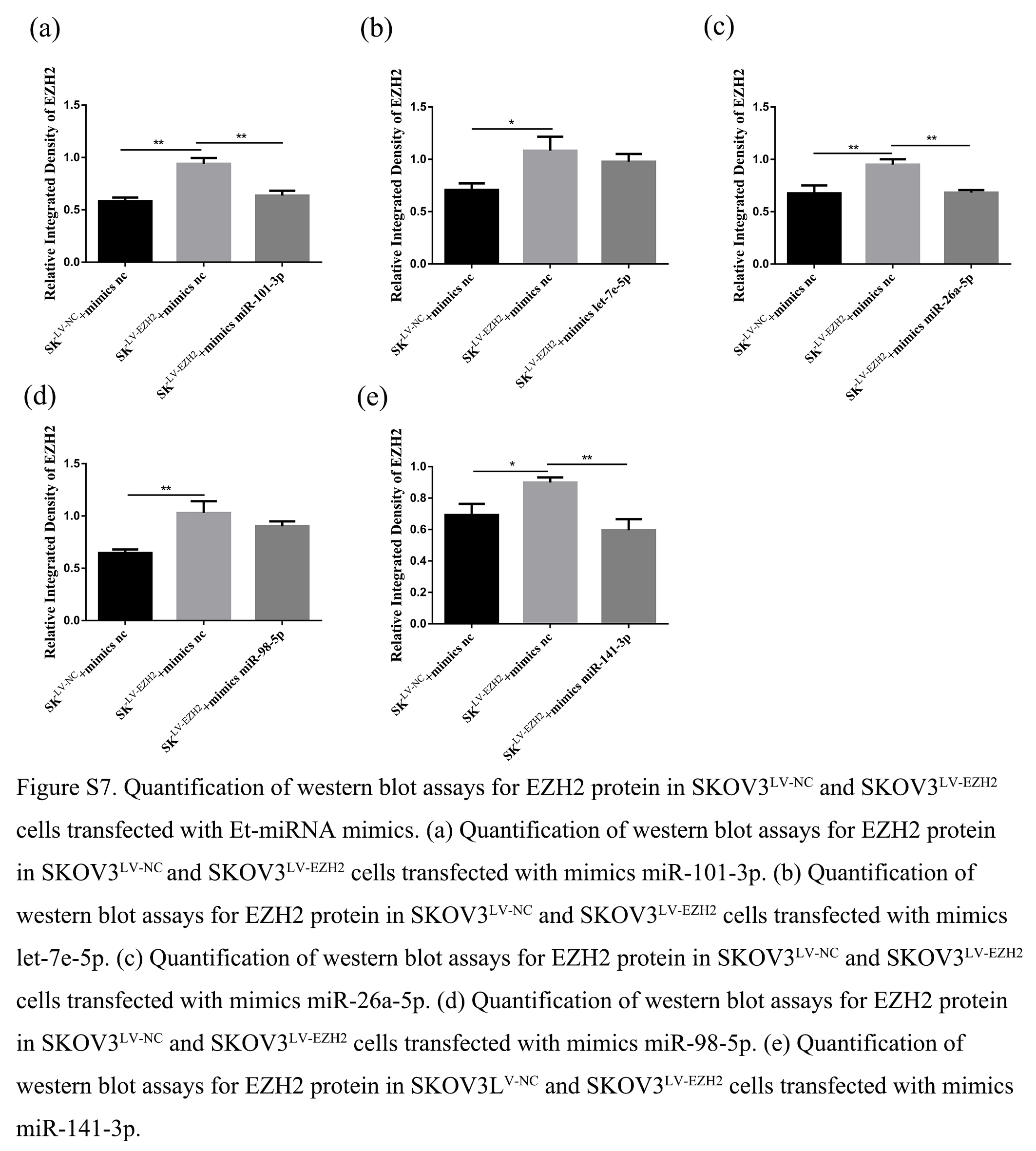

Supplement: Supplementary file 7 [file Image_7.jpeg]
